# *AtOPR3* specifically inhibits primary root growth in Arabidopsis under phosphate deficiency

**DOI:** 10.1038/srep24778

**Published:** 2016-04-22

**Authors:** Hongyan Zheng, Xiaoying Pan, Yuxia Deng, Huamao Wu, Pei Liu, Xuexian Li

**Affiliations:** 1 Department of Plant Nutrition, China Agricultural University, Beijing, 100193, China; 2 Department of Ecology, China Agricultural University, Beijing, 100193, China

## Abstract

The primary root plays essential roles in root development, nutrient absorption, and root architectural establishment. Primary root growth is generally suppressed by phosphate (P) deficiency in *A. thaliana*; however, the underlying molecular mechanisms are largely elusive to date. We found that *AtOPR3* specifically inhibited primary root growth under P deficiency via suppressing root tip growth at the transcriptional level, revealing an important novel function of *AtOPR3* in regulating primary root response to the nutrient stress. Importantly, *AtOPR3* functioned to down-regulate primary root growth under P limitation mostly by its own, rather than depending on the Jasmonic acid signaling pathway. Further, *AtOPR3* interacted with ethylene and gibberellin signaling pathways to regulate primary root growth upon P deficiency. In addition, the *AtOPR3*’s function in inhibiting primary root growth upon P limitation was also partially dependent on auxin polar transport. Together, our studies provide new insights into how *AtOPR3*, together with hormone signaling interactions, modulates primary root growth in coping with the environmental stress in *Arabidopsis*.

Initiated during embryo development, the primary root is the fundamental part of a root system that absorbs mineral nutrients and provides mechanical support for shoot growth. The primary root plays important roles in nutrient uptake during the early period of plant development and displays a surprising capacity of nutrient uptake in the later developmental stage, too. The maize *rtcs* (rootless for crown and seminal roots) mutant only with a functional primary root is able to finish its life cycle and generates progeny as a normal plant does^1^, suggesting that the primary root, with great growth plasticity in response to internal and external stimuli, is sufficient to support whole plant growth in terms of nutrient and water uptake. Root growth adapts to environmental changes in soil composition, and water and mineral nutrient availability via developmental and configurational alterations^2^. In the agricultural ecosystem, nutrient insufficiency becomes a major limiting factor for plant growth, development, and productivity, which, together with intrinsic developmental programs, reshapes root architectural patterning for nutrient favorable root morphogenesis^3^.

Phosphorus (P) deficiency is a very common abiotic stress that inhibits plant growth and reduces crop productivity due to poor mobility and low availability of phosphate in soils^4^. In contrast to inconsistent effects of low P on primary root growth in different maize inbred lines^5^,^6^,^7^, low P inhibits cell division in the meristematic region and promotes premature cell differentiation within the root tip, resulting in severe suppression of primary root growth in *Arabidopsis*^8^,^9^. Several genes have been reported to be involved in mediating primary root responses to low P in *Arabidopsis*. The PHOSPHATE DEFICIENCY RESPONSE 2 gene (*PDR2*) encodes a P5-type ATPase regulating expression of SCARECROW (*SCR*), a key regulator of root patterning and stem-cell niche maintenance in roots under P deficiency, and the absence of PDR2 protein further reduced primary root growth under the low P condition^10^,^11^. The other two genes, *PLDζ(1,2)* (Phospholipase Ds) and *PRD* (Phosphate root development), also positively regulate primary root growth under P deficiency^12^,^13^, while *LPR* (Low phosphate root) has a negative regulatory role^14^. Interestingly, both *PDR2* and *LPR1* are expressed in the stem-cell niche and distal root meristem and collaboratively modulate root meristem activities in response to external P in an ER-resident pathway^11^. Beyond these regulators, hormones play critical roles in root patterning under low P conditions. Ethylene modulates cell division in the quiescent center during root development^15^ and plays a role in restricting primary root growth in response to low P in *Arabidopsis*^9^. P deficiency can also lead to lower concentrations of bioactive gibberellins (GA) that may promote DELLA protein accumulation which, in turn, restricts primary root growth in *Arabidopsis*^16^. Exogenous application of GA can restore primary root growth in *Arabidopsis* under low P conditions, while DELLA-deficient mutants are less responsive to P deficiency in terms of primary root growth^16^. Different from ethylene and GA signaling, auxin regulates root growth under low P conditions via its redistribution^17^. Higher auxin concentrations in the root meristem caused by *HPS4* (*hypersensitive to phosphate starvation 4*) mutation or blockage of auxin polar transport by 2,3,5-triiodobenzoic acid (TIBA) inhibits primary root elongation in Arabidopsis under P deficiency^18^,^19^.

Jasmonic acid (JA), a vital hormone mediating plant defense and development^20^,^21^,^22^,^23^,^24^, has no molecular link with primary root growth suppression in *Arabidopsis* under P deficiency, although exogenous application of JA is able to suppress primary root growth of Arabidopsis seedlings by reducing root meristematic activity and promoting abnormal quiescent center division under sufficient P conditions^9^,^25^,^26^,^27^. *AtOPR3* is the only gene responsible for JA biosynthesis among six *OPR* genes in *Arabidopsis*^28^,^29^,^30^. Loss-of-function of *AtOPR3* or its maize ortholog causes male sterility which is reversible by JA spray^23^,^28^, revealing a vital role of *AtOPR3* in mediating flower development. Interestingly, enhanced primary root growth under low P stress in the *lpi4* (low P insensitive 4) mutant is correlated with down-regulation of *AtOPR3* expression^9^. This correlation, together with a recent report that *AtOPR3* is involved in lateral root development^31^, implies that *AtOPR3* may be a potential player regulating primary root growth in P-deficient *Arabidopsis*. In spite of above advances, molecular and genetic mechanisms of growth suppression of the primary root by P limitation are still largely elusive. It is particularly interesting to investigate the potential functions of *AtOPR3*, if any, in regulating primary root growth in *Arabidopsis* upon P deficiency and to reveal the underlying molecular mechanisms. Considering the limitation of P resources and environment pressure of P fertilization^32^,^33^, it is also economically imperative to explore adaptive mechanisms of plants with insufficient P supplies.

## Results

### *AtOPR3* knockout plants had a longer primary root than WT seedlings only under the low P condition among three macronutrient deficiencies

**Figure 1:**
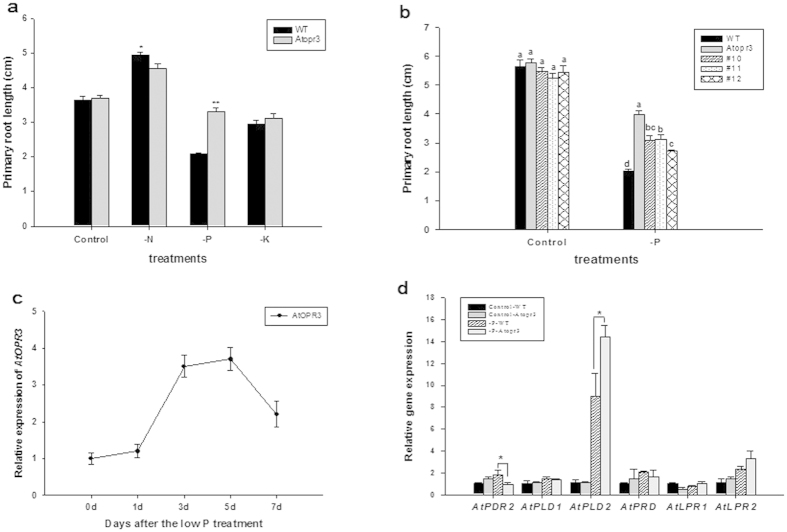
*AtOPR3* specifically inhibited primary root growth in Arabidopsis under P deficiency. (**a**) Five-day-old seedlings were transferred to full nutrient (Control), low N (-N), low P (-P), or low K (-K) conditions respectively for 7 days. WT, wild type; *Atopr3*, the mutant line. Results were presented as means (n = 30) with error bars (standard deviation), and asterisks indicated significant differences as determined by a t-test analysis (*P < 0.05; **P < 0.01). (**b**) Comparision of primary root length among WT, *Atopr3* and three representative *AtOPR3* complementary lines (#10, #11 and #12). Results were presented as means (n = 30) with error bars (standard deviation), and different letters indicated significant differences between different lines within the same treatment (P < 0.05). (**c**) The relative expression level of *AtOPR3* in WT during a 7-day low P treatment. Primary roots were sampled at 0d, 1d, 3d, 5d and 7d after transfer, and expression levels of *AtOPR3* were determined by RT-qPCR. Data represented as means and SD (standard deviation) of three independent biological replicates. (**d**) Relative expression levels of six known genes regulating root response to low P in WT and *Atopr3* mutant plants under the whole nutrient (Control) or low P (-P) treatment. Root samples were harvested 7d after transfer, and mRNA abundance was determined by RT-qPCR. Error bars represented SD of three independent biological replicates.

Roots respond to three macronutrient deficiencies via distinct morphological modifications. In *Arabidopsis*, low nitrogen (N) or P stimulates overall root growth to enhance nutrient uptake, while low potassium (K) suppresses entire root growth^34^,^35^,^36^. Within a root system, low N promotes lateral root growth with little effect on primary root growth^36^; whereas low P hinders primary root growth and induces compensatory growth of lateral roots^34^,^37^. In our results, primary root growth was inhibited by low P or K supply in sharp contrast to a significant stimulatory effect of low N. Primary root length under low N, P, and K was respectively 1.4, 0.6, 0.8 times as that of control plants (Table 1). Surprisingly, *AtOPR3* knockout mutants had a 40% longer primary root than wild type (WT) plants under the low P condition (Fig. 1a, Table 1). Primary roots of *Atopr3* plants remained suppressed under the low K condition and showed no significant difference compared with WT plants (Fig. 1a). These data suggested that *AtOPR3* is specifically required to inhibit primary root growth under P deficiency.

To confirm that enhanced primary root growth in *Atopr3* under low P was indeed due to *AtOPR3* knockout, the *AtOPR3* coding sequence was expressed in the *Atopr3* mutant driven by the *AtUbiquitin* promoter. Three independent transgenic lines were chosen for phenotypic analysis. As expected, transgenic plants showed reduced primary root growth compared to the *Atopr3* mutant (Fig. 1b). Notably, gene transformation was unable to fully restore the inhibitory effect probably due to imperfect drive of a non-native promoter.

To better understand whether *AtOPR3* mediates primary root growth at the transcriptional level, we analyzed relative abundance of *AtOPR3* transcripts over a 7-day low P treatment. Low P stimulated *AtOPR3* expression with a clear peak on day 5 after the treatment, followed by a gradual decline to the control level in WT seedlings (Fig. 1c). The low P responsive expression curve of *AtOPR3* revealed that *AtOPR3*, as a negative regulator, may hamper primary root growth via transcriptional regulation. Notably, P1BS and P1BS-like elements are PHR1 (Phosphate Starvation Response 1)/PHLs (PHR1-Like)-bound sequences involved in regulation of P deficiency responses^38^,^39^,^40^. We found one putative P1BS element (GAATATAC_-1897_) and one putative P1BS-like (AAATATCC_-910_) element in the 5′-upstream region of *AtOPR3* (Fig. S1). We further analyzed expression levels of previously reported genes (*PDR2, PLDζ1, PLDζ2, PRD, LPR1* and *LPR2*) regulating primary root growth in response to P deficiency, and found down-regulation of *PDR2* expression and up-regulation of *PLDζ2* expression in the *Atopr3* mutant root under P deficiency (Fig. 1d), although there was no significant difference in transcript accumulation of *LPR1*, *LPR2*, *PRD*, *and PLDζ1* between WT and *Atopr3* seedlings under the same condition (Fig. 1d).

### Morphological analysis of primary roots showed that *AtOPR3* inhibited elongation growth of the root tip

**Figure 2:**
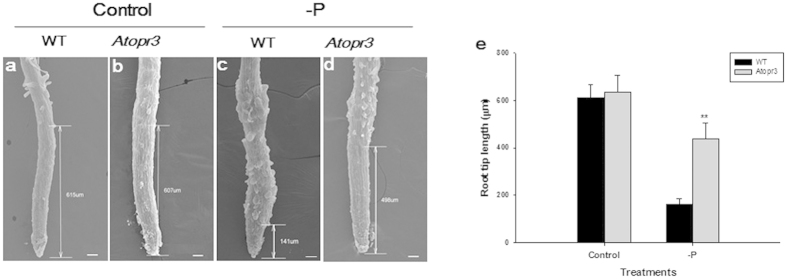
*AtOPR3* negatively mediates primary root growth via inhibiting longitudinal growth of the root tip. (**a–d**) Scan electron microscopy analysis of root tip length in WT and *Atopr3* mutant plants under the whole nutrient (Control) or low P (-P) treatment. (**e**) Statistical analysis of root tip length between WT and *Atopr3* mutant plants. Results were presented as means (n = 10) with error bars (standard deviation), and asterisks indicated significant differences as determined by a t-test analysis (**P < 0.01).

Longitudinal growth of the root tip is a prerequisite for fast root growth^41^. To investigate whether *AtOPR3* modulates root tip growth, we analyzed in-depth morphological variation in root tips using scanning electron microscopy (Fig. 2a–d). In WT plants, root tip growth was suppressed by P limitation (Fig. 2a,c,e), and average root tip length (from the very root tip to the position where the first root hair emerges) was reduced from 613 (±52, n = 10) μm to 164 (±22, n = 10) μm. Root tip length (635 ± 70, n = 10) of *Atopr3* mutant plants was similar to that of WT plants with sufficient P supply (Fig. 2a,b,e). However, *Atopr3* mutant plants had approximately 2.7-fold long root tips (437 ± 66, n = 10) compared to those of WT plants (164 ± 22, n = 10) under P deficiency (Fig. 2c–e), indicating that *AtOPR3* negatively mediates primary root growth, at least partially, via inhibiting longitudinal growth of the root tip.

### Exogenous application of JA or JA inhibitors did not eliminate the significant difference in primary root growth between WT and *Atopr3* mutant plants under P deficiency

**Figure 3:**
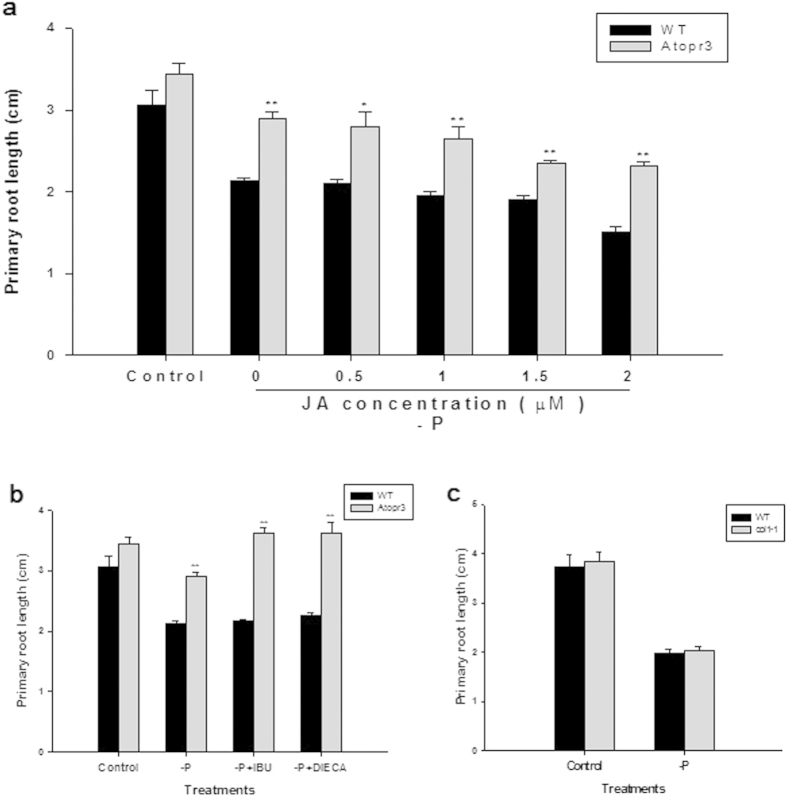
*AtOPR3*’s function in mediating primary root growth under low P is independent of JA biosynthesis and signaling. Primary root length of wild type and mutant lines (*Atopr3* and Ws; coi1-1 and Col-0) were analyzed under various treatments. Five-day-old seedlings were transferred to whole nutrient (Control) or low P (-P) solutions in the presence or absence of various chemicals for 7 days. IBU and DIECA were used to block JA biosynthesis. (**a**) Effects of various concentrations of JA on primary root growth. (**b**) Effects of 5 μM IBU (-P + IBU) or 5 μM DIECA (-P + DIECA) on primary root growth. (**c**) Comparison of primary root growth between Col-0 and coi1-1 under control and low P conditions. Results were presented as means (n = 30) with error bars (standard deviation). Asterisks indicated significant differences as determined by a t-test analysis (*P < 0.05; **P < 0.01).

Given that *AtOPR3* is a critical enzyme for JA biosynthesis, *AtOPR3* may mediate root growth under low P conditions via the JA signaling pathway. We treated plants under P deficiency with exogenous JA to reduce primary root growth in *Atopr3* plants. Primary roots of *Atopr3* plants were still 1.3 times longer than that of WT plants although JA application reduces primary root growth in WT and *Atopr3* mutants at different ratios (Table 1). The primary root is 19.0% shorter in *Atopr3* mutants and 10.3% shorter in WT plants after the JA treatment (Table 1), indicating a larger effect of the JA treatment on primary root growth of the *Atopr3* mutant plants. Next, we adjusted JA concentrations in a reasonable range to further minimize the length difference in the primary root of the *Atopr3* mutant and WT plants under P deficiency. However, the JA treatment at other concentrations failed to eliminate the significant difference in primary root length between *Atopr3* mutant and WT plants, either (Fig. 3a). We then applied JA biosynthesis inhibitors ((S)-(+)-Ibuprofen, IBU and Diethyldithiocarbamic acid, DIECA) to remove restriction of primary root growth presumably exerted by *AtOPR3* mediated JA synthesis and signaling in WT plants under P deficiency. Unexpectedly, JA biosynthesis inhibitor IBU stimulated 24.8% more primary root growth in *Atopr3* mutant plants compared to non-IBU treated mutant plants, by contrast to only 1.9% stimulation in WT plants (Fig. 3b, Table 1). Another JA biosynthesis inhibitor DIECA also had a larger stimulatory effect on primary root growth of *Atopr3* mutant plants as compared to that in WT plants (Fig. 3b, Table 1). Together, these results suggested that the *AtOPR3*’s function in negatively mediating primary root growth under P deficiency is likely independent of JA biosynthesis.

To further differentiate the short primary root phenotype of *Atopr3* under P deficiency from JA signaling, we took advantage of the *coi1-1* (*coronatine insensitive 1*) mutant line to analyze whether this mutant line has a longer primary root under P deficiency. In contrast to an obviously longer primary root in the *Atopr3* mutant line than that in WT plants upon P deficiency, *coi1-1* mutant plants had as short primary roots as WT plants (Fig. 3c), suggesting that blockage of JA signaling itself is not able to promote primary root growth either with sufficient P supply or under P deficiency.

### *AtOPR3* interacted with ethylene signaling to mediate primary root growth under the low P condition

**Figure 4:**
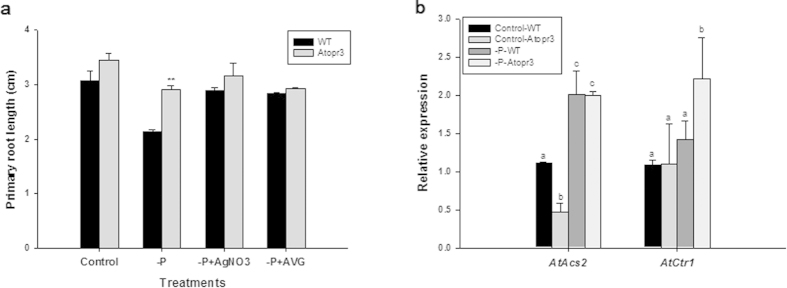
The role of ethylene signaling in mediating primary root growth in *Atopr3* and WT plants. Seedlings were grown as described in Fig. 3. (**a**) Effects of 5 μM AgNO_3_ (-P + AgNO_3_) or 1.25 μM AVG (-P + AVG) on primary root growth. Results were presented as means (n = 30) with error bars (standard deviation). Asterisks indicated significant differences as determined by a t-test analysis (**P < 0.01). (**b**) Relative expression levels of critical genes mediating ethylene biosynthesis or signaling (determined by RT-qPCR) in WT and *Atopr3* mutant plants. Data represented means and SD of three independent biological replicates. Different letters indicated means with significant differences (P < 0.05).

Transcriptomic analysis shows that expression of genes controlling ethylene biosynthesis are up-regulated in Arabidopsis under low P, and inhibition of ethylene biosynthesis is able to maintain normal meristem organization and activity in low P growth medium^9^,^27^,^42^. To investigate whether *AtOPR3* interacts with ethylene signaling in regulating primary root growth in response to P deficiency, inhibitors of ethylene signaling (AgNO_3_) and biosynthesis (Aminoethoxyvinyl glycine hydrochloride, AVG) were separately applied into growth media. Both AgNO_3_ and AVG treatments had a larger stimulatory effect on primary root growth in WT plants as compared to that in the *Atopr3* mutant (Table 1). Primary root length of WT plants treated with AgNO_3_ increased to 1.4 times when compared to non-AgNO_3_-treated plants under P deficiency, successfully closing the length gap between primary roots of WT and *Atopr3* mutant plants (Fig. 4a, Table 1). AVG addition also nearly restored primary root growth of WT plants under P deficiency, making it statistically indistinguishable from that of *Atopr3* plants (Fig. 4a, Table 1). These results indicated that *AtOPR3* likely interacts with ethylene signaling to control primary root growth in response to P limitation.

Ethylene biosynthesis is regulated by ACC synthase (ACS), a rate-limiting enzyme that catalyzes synthesis of the ethylene precursor ACC^43^. Downstream of ethylene signaling transduction is a Raf-like Ser/Thr kinase CTR1 that negatively regulates ethylene signaling^44^. We analyzed the expression level of *AtACS2* and *AtCTR1* to further characterize molecular interaction of *AtOPR3* with ethylene signaling. Reverse transcription-quantitative real time PCR (RT-qPCR) analysis showed no significant difference in the expression level of *AtACS2* between WT and mutant plants (Fig. 4b), whereas the expression level of *AtCTR1* in the *Atopr3* mutant was 1.5-fold as that in WT plants under P deficiency (Fig. 4b), suggesting that *AtOPR3* may interact with ethylene signaling by restraining up-regulation of *AtCTR1* expression in WT plants under P deficiency.

### The GA signaling pathway was also involved in *AtOPR3*-mediated primary root growth under P deficiency

**Figure 5:**
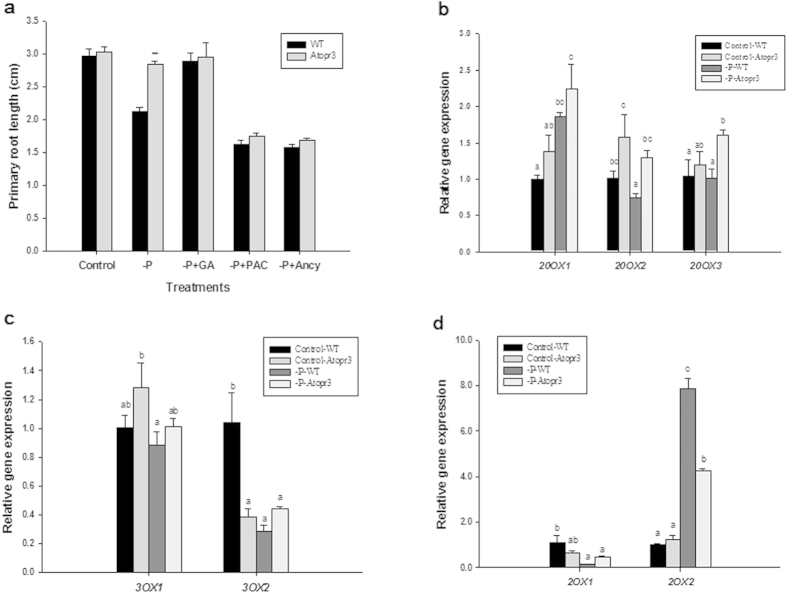
The role of GA signaling in regulating primary root growth in *Atopr3* and WT plants. Seedlings were grown as described in Fig. 3. PAC and Ancy were applied to inhibit GA biosynthesis. (**a**) Effects of 7.5 μM GA (-P + GA), 7.5 μM PAC (-P + PAC), 5 μM Ancy (-P + Ancy) on primary root growth. Results were presented as means (n = 30) with error bars (standard deviation). Asterisks indicated significant differences as determined by a t-test analysis (**P < 0.01). (B-D) Relative expression levels of genes mediating GA biosynthesis (determined by RT-qPCR) in WT and *Atopr3* mutants. (**b**) GA 20-oxidases, (**c**) GA 3-oxidases, and (**d**) GA 2-oxidases. Data represented means and SD of three independent biological replicates. Different letters indicated means with significant differences (P < 0.05).

GA is a key player regulating root development and growth under P limitation^16^. To detect the potential interaction of *AtOPR3* with the GA signaling pathway, various concentrations of GA and GA biosynthesis inhibitors (Paclobutrazol, PAC and Ancymidol, Ancy) were separately applied to growth media (Table S1). Under low P conditions, GA application promoted more primary root growth in WT plants than in *Atopr3* plants so that primary root length had no significant difference between the mutant and WT plants (Fig. 5a, Table 1); GA inhibitor (PAC and Ancy) also eliminated the length gap between WT and *Atopr3* plants (Fig. 5a). These results suggested that regulation of primary root growth by *AtOPR3* under P deficiency is dependent on GA signaling.

The bioactive GA level is modulated by transcriptional up-regulation of GA 20-oxidases (*GA20OX*) and GA 3-oxidases (*GA3OX*) or transcriptional down-regulation of GA 2-oxidases (*GA2OX*)^45^,^46^,^47^. We analyzed expression levels of *GA20OX*, *GA3OX*, and *GA2OX* via RT-qPCR to further characterize molecular interaction of *AtOPR3* with GA metabolism. We found significantly higher expression levels of *GA20OX2 and GA20OX3* in *Atopr3* than in WT plants under low P supply (Fig. 5b). There was no significant difference in *GA3OX* transcription between WT and *Atopr3* mutant plants under P limitation, in spite of the higher expression level of *GA3OX2* in WT plants with sufficient P supply (Fig. 5c). On the other hand, *Atopr3* mutants had a significantly lower expression level of *GA2OX2* than WT plants under low P conditions (Fig. 5d). Taken together, *Atopr3* mutants had more GA biosynthesis and less degradation, resulting in higher bioactive GA levels in the mutant line than in WT plants under low P conditions, which stimulated root growth under P limitation.

In addition, auxin plays an essential role in controlling root growth via asymmetric distribution^48^. Inhibition of auxin polar transport by TIBA reduced primary root growth by 33.0% and 23.0% respectively in *Atopr3* mutant and WT plants under P deficiency compared to non-TIBA-treated plants, implying that *Atopr3* mutant plants are likely more sensitive to TIBA than WT seedlings (Table 1, Fig. S2). However, the *Atopr3* plants still had significantly longer primary roots than WT seedlings regardless of various concentrations of TIBA treatments (Table S1). Although auxin preconditions root growth, our data showed that auxin signaling was not a major player in *AtOPR3* mediated primary root growth under P deficiency.

## Discussion

The primary root plays essential roles in nutrient uptake, and is sufficient for plants to finish their lifecycle^1^. Low P bioavailability in the soil makes P deficiency one of the most limiting factors for fast plant growth, reproduction, and food production^32^. A conspicuous change in P deficient *Arabidopsis* is arrested primary root growth^34^. Although several genes and hormone signaling have been reported to be involved in regulating primary root growth upon P deficiency^49^, molecular and genetic mechanisms of growth inhibition of the primary root by P deficiency in *Arabidopsis* remain fundamental questions to be elucidated in plant stress physiology.

### *AtOPR3* specifically inhibits primary root growth under the low P condition by restraining longitudinal growth of the root tip in *Arabidopsis*

Macronutrient deficiencies alter root architecture in certain common ways in Arabidopsis. P and K deficiencies inhibit primary root growth, and N and P deficiencies enhance lateral root growth^34^,^35^,^36^. It is particularly important to dissect these commonalities and identify nutrient-specific features at the molecular level. *AtOPR3*, as a critical enzyme in JA biosynthesis, has crucial biological functions in flower development and defense response^28^,^50^. Here, *Atopr3* mutant plants showed compensatory primary root growth under N deprivation and no effect on primary root growth under K privation compared to WT plants under the same condition (Fig. 1a). Importantly, the primary root of *Atopr3* plants showed continuous growth, rather than arrested by P deficiency as shown in WT plants (Fig. 1a). Our results demonstrated a novel function of *AtOPR3* in regulating primary root response to abiotic stresses: *AtOPR3* inhibited primary root growth only under P deficiency among three macronutrient deficiencies (Fig. 1a).

Up-regulation of *AtOPR3* expression in wild-type plants under P deficiency confirmed that it is a negative regulator functioning at the transcriptional level (Fig. 1c). The presence of the P1BS and P1BS-like elements in the *AtOPR3* 5′-upstream region indicates that *AtOPR3* could potentially interact with PHR1/PHLs in regulation of root responses to P deficiency^38^,^39^,^40^ (Fig. S1). Many genes are common regulators in root response to N, P, and K deficiencies. Identification of *AtOPR3* as a P specific root growth regulator not only helps interpret contrasting performance of the primary root under macronutrient deficiencies, but also provides a powerful molecular marker in P nutritional diagnosis. Further, *AtOPR3* negatively regulates primary root growth partially by inhibiting longitudinal growth of the root tip (Fig. 2a–e). We speculated that the inhibitory effect of *AtOPR3* on primary root growth was due to a dramatic decrease either in cell number or cell length in elongation and apical meristematic zones. This is consistent with previous report that low P inhibits primary root growth in *Arabidopsis* through arresting cell division and promoting cell differentiation in these two zones^8^. Six genes (*PDR2*, *PLDζ1*, *PLDζ2, PRD*, *LPR1* and *LPR2*) are previously reported to modulate primary root growth under P deficiency^10^,^11^,^12^,^13^,^14^,^51^; however, four (*PLDζ1, PRD*, *LPR1*, *LPR2*) of them had no significantly altered expression in *Atopr3* mutant plants upon P deficiency (Fig. 1d). Down-regulation of *PDR2* is expected to have a negative effect on primary root growth^10^,^11^, in contrast to stimulatory root growth in the *Atopr3* mutant plant under low P; 1.5-fold up-regulation of *PLDζ2* expression probably causes no effect on root growth given that only simultaneous knockout of *PLDζ1* and *PLDζ2* leads to a shorter primary root^12^. Thus, we concluded that *AtOPR3* inhibits primary root growth under the low P condition likely independent of these reported molecular pathways, revealing a novel molecular mechanism of root growth regulation in *Arabidopsis* in response to the P stress.

### The *AtOPR3*’s function in regulating primary root growth under low P conditions is mostly independent of JA signaling

*AtOPR3* regulates flower development and pathogen defense, and *AtOPR3* knockout causes male sterility and seriously weakens plant resistance to pathogen attack^23^,^28^. These functional defects in the *Atopr3* mutant line are fully restored by exogenous JA application, suggesting that *AtOPR3* functions via the JA signaling pathway. Similarly, *AtOPR3* down-regulates primary root growth under P deficiency probably via JA signaling, too. However, under P deficiency, JA addition was unable to suppress compensatory growth of the primary root in the *Atopr3* mutant, and JA inhibitors failed to restore primary root growth of WT plants (Fig. 3a,b), clearly suggesting that stimulated primary root growth in the *Atopr3* mutant under P deficiency is primarily not a major consequence of down-regulation of *AtOPR3* mediated-JA synthesis, but a more direct result derived from functional knockout of *AtOPR3* itself. Further, the JA signaling mutant *coi1-1* exhibited growth arrest of the primary root in response to low P just as WT seedlings did (Fig. 3c), providing strong genetic evidence that *AtOPR3* down-regulates primary root growth under low P conditions mostly independent of the JA signaling pathway, similar to many other genes with multiple functions depending on biological contexts. TRH1 primarily functions as a potassium transporter, mediates auxin transport, and is required for morphogenesis of root hairs^52^,^53^,^54^. The potassium transporter KUP2 is also essential for cell expansion in the shoot^55^. Although exogenous JA application inhibits primary root growth to different extents in *Arabidopsis*^26^, our data implied that JA is not a dominant player in *AtOPR3* mediated primary root growth in response to P deficiency. However, we are not ruling out the possibility that JA may function in this process via unidentified interactions with other hormone signaling pathways.

### *AtOPR3* interacts with ethylene and GA signaling pathways to regulate root growth under low P conditions

Although it is well established that ethylene plays an essential role in modulating root development^15^, it remains largely unclear how ethylene is involved in regulation of primary root growth under P deficiency. Upregulated expression of ethylene biosynthesis and signaling related genes under low P supports a potential role of ethylene in mediating plant response to P limitation^42^, which is further strengthened by the recent finding that AVG and AgNO_3_ treatments are able to maintain meristem organization and activity under P deficiency, while ACC addition disrupts meristem functions in the primary root under low or high P supply^9^. In our study, ethylene signaling and biosynthesis inhibitors fully restored primary root growth in WT and *Atopr3* plants under low P conditions and closed the large length gap in primary roots between WT and mutant plants (Fig. 4a). Thus, we speculated that *AtOPR3* inhibited primary root growth via interacting with the ethylene signaling pathway in *Arabidopsis*. We further found that CTR1, negatively regulating ethylene signaling as a downstream receptor^44^,^56^, had significantly higher expression levels under low P in the *Atopr3* mutant seedlings than in the WT plants (Fig. 4b). Up-regulation of *AtCTR1* expression in *Atopr3* mutant plants alleviated inhibitory effects of P-deficiency triggered ethylene signaling on primary root growth.

Not just ethylene signaling, the decrease in bioactive GA levels and subsequent DELLA accumulation under low P conditions result in growth arrest of the primary root and trigger other P starvation responses^16^. In our studies, GA inhibitors (PAC and Ancy) had a larger inhibitory effect on primary root growth of *Atopr3* plants than on WT, and exogenous GA promoted more primary root growth in WT plants than in *Atopr3* mutants under low P conditions (Fig. 5a). Either treatment eliminated the significant difference in primary root length between the mutant and WT plants exerted by the P stress, suggesting that *AtOPR3* also interacts with GA signaling to mediate primary root growth in response to P limitation. In P-deficient WT plants, *AtOPR3* may down-regulate accumulation of bioactive GAs to reduce primary root growth, putatively via down-regulating *GA20OX2* and *GA20OX3* transcription and up-regulating *GA2OX2* transcription (Fig. 5b,d), although specific mechanisms need further investigation. Functional knockout of *AtOPR3* may result in higher bioactive GA levels that in turn promote root growth in adaptation to external stimulus of P stress (Fig. 5a,b,d).

Although auxin is also an essential growth regulator, and exogenous auxin application restricts primary root growth^17^,^57^, blockage of auxin polar transport by TIBA failed to eliminate the significant length difference between WT and *Atopr3* mutant plants in our studies (Table 1, Fig. S2). Therefore, we conclude that *AtOPR3* mostly interacts with ethylene and GA signaling pathways to modulate root growth, with auxin signaling as a secondary interaction when coping with the P stress.

**Figure 6:**
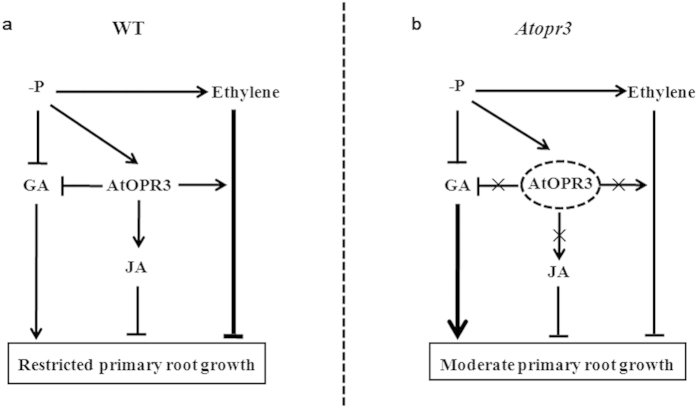
A schematic model of how *AtOPR3* regulates primary root growth under low P conditions, putatively via interacting with ethylene and GA signaling pathways in Arabidopsis. When supplied with sufficient P, both WT and *Atopr3* mutant plants show normal root growth due to absence of low P stress signals. (**a**) In the WT plants, *AtOPR3* expression is stimulated by low P supply. Up-regulation of *AtOPR3* causes three biological consequences: stimulation of JA biosynthesis, enhancement of ethylene signaling, and down-regulation of the bioactive GA content, which collectively suppresses primary root growth under P deficiency. (**b**) In *AtOPR3* knockout mutant plants, absence of functional *AtOPR3* transcripts blocks JA biosynthesis, reduces strength of P-stress directed ethylene signaling, and indirectly up-regulates the level of bioactive GA. All these alterations result in continuous primary root growth as a whole in spite of P limitation.

Therefore, we proposed a mechanistic model of how *AtOPR3* mediates primary root growth via interaction with ethylene and GA signaling pathways (Fig. 6). With sufficient P supply, the primary root maintains its normal growth rhythm in WT plants; blockage of JA biosynthesis itself has no any inhibitory or significant stimulatory effect on primary root growth in the *Atopr3* mutant (Fig. 3b,c). Under P limitation, up-regulation of *AtOPR3* expression in the WT plants promotes JA biosynthesis, enhances P stress-triggered ethylene signaling potentially via down-regulating *CTR1* expression, and attenuates GA signaling by reducing bioactive GA contents, which collectively suppresses primary root growth (Fig. 6a). Although our observation did not support that JA is a major player in *AtOPR3* mediated-primary root growth (Fig. 3), an increase in JA content, caused by up-regulation of *AtOPR3* expression, may reduce root growth to a certain extent or has other complicated effects via unknown hormone signaling interactions. By contrast, knockout of *AtOPR3* in the mutant line indirectly up-regulates the level of bioactive GA, reduces strength of P-stress directed ethylene signaling, and terminates JA biosynthesis under P deficiency (Fig. 6b). All these modulations alleviate suppression effects of the P stress on primary root growth, resulting in a significantly longer primary root in the *Atopr3* mutant plants than in WT plants, although underlying molecular mechanisms remain to be further explored.

## Materials and Methods

### Materials

Arabidopsis ecotype Wassilewskija (*Ws*), Columbia (*Col*), T-DNA insertional mutant *Atopr3*^28^ in which *AtOPR3*-mediated JA biosynthesis is blocked, and *coi1-1* mutant^58^ in which JA signaling is blocked were used in our experiments.

### Plasmid construction and plant transformation

*AtOPR3* coding sequence was cloned into the T-vector pMD19 (Takara), and then inserted into a pUT-hyg vector^59^ (with the *AtUbiquitin* promoter and hygromycin resistance) using restriction sites SalI and SpeI for functional complementation. The resulting plasmid was transformed into *Agrobacterium tumefaciens* GV3101 by electroporation and further delivered into the *Atopr3* plants using the standard floral dip method^60^. Homozygous lines of the T3 generation were used for phenotypic analysis.

### Arabidopsis growth

Seeds were first imbibed in water in the dark at 4 °C for 2 days to break dormancy. Then seeds were surface sterilized (75% ethanol (V/V) for 1 minute and 2% NaClO (V/V) for 2 minutes, followed by six rinses in sterile water) and sown on 1/2MS (Murashige and Skoog Stock) plate with 1% sugar and 0.8% agar. Plates were placed vertically in a standard plant growth chamber (Kooland, China) under the following condition: 22 °C, illumination 100 μmol photons m^−2^ s^−1^, 16/8 h light/dark, 60% relative humidity. For *coi1-1* mutant screen, seeds were sown on the plate with 25 μM Methyl jasmonate for three days to screen for homozygous plants which were then transferred to 1/2 MS for continuous growth. After five days, the uniform seedlings were transferred to plates with whole nutrient, N deficient (5 μM N), P deficient (10 μM P) or K deficient MS medium (5 μM K) respectively. The whole nutrient medium contained 21 mM NH_4_NO_3_, 19 mM KNO_3_, 1.25 mM KH_2_PO_4_, 3 mM CaCl_2_, 1.5 mM MgSO_4_·7H_2_O, 0.005 mM KI, 0.1 mM MnSO_4_·H_2_O, 0.03 mM ZnSO_4_·H_2_O, 0.001 mM Na_2_MoO_4_·2H_2_O, 0.0001 mM CuSO_4_·5H_2_O, 0.0001 mM CoCl_2_·6H_2_O, 0.1003 mM H_3_BO_3_, 0.1 mM EDTA-Fe. The pH maintains at 5.8. To make the low N and low P media, NH_4_NO_3_, KNO_3_, KH_2_PO_4_ were replaced by KCl in the nutrient solution. To make low K media, KNO_3_ and KH_2_PO_4_ were replaced by NH_4_H_2_PO_4_ in the nutrient solution.

The plates were supplemented with or without various hormones or inhibitors. All experiments had six technical replicates, five biological replicates. Wild-type and mutant plants were transferred and grown on different sides of the same plate. Seedlings were harvested seven days after transfer. For each treatment, primary roots of 30 12-day-old seedlings were measured from the root tip to the hypocotyl base with a ruler.

### Hormone treatments

Before making plates, appropriate amount of individual hormone solutions were added into the culture medium around 50 °C. Jasmonic acid (Sigma), (S)-(+)-Ibuprofen (Sigma), Paclobutrazol (Sigma), Gibberellic acid (Sigma), Ancymidol (Sigma) were dissolved in ethanol. Aminoethoxyvinyl glycine hydrochloride (Sigma), AgNO_3_ (Sinopharm Chemical Reagent Co., LTD), 2,3,5-triiodobenzoic acid (Sigma) and Diethyldithiocarbamic acid (Sigma) were dissolved in ddH_2_O.

### Transcriptional analyses

Root samples were quickly harvested and immediately frozen in liquid nitrogen. Arabidopsis total RNA was extracted with the RNAprep pure Plant kit (TIANGEN, Beijing). cDNA synthesis and RT-qPCR was carried out following manufacturer’s instructions^61^. The *Arabidopsis TUB4* gene was used as a positive internal control. The primers used for RT-qPCR analysis and gene cloning are listed in Table S2.

### Preparations of Arabidopsis root samples for scanning electron microscopy (SEM)

We followed a standard protocol slightly modified after Burgess and Linstead^62^. Briefly, 0.5 mm root tip samples were fixed with 2.5% Glutaraldehyde and 1% osmic acid sequentially. After wash in PBS (PH 7.2, 0.1M) buffer, fixed root samples were dehydrated with six gradient ethanol (30%–50%–70%–80%–90%–100%) and isoamyl acetate three times, followed by critical-point drying (HITACHI HCP-2) and ion sputtering (EIKO IB-3). The samples were than analyzed on the scanning electron microscope (HITACHI S-3400N) according to the standard instructions. The root tip referred to the section from the very tip point to the point where the first root hair primordium initiated.

## Additional Information

**How to cite this article**: Zheng, H. *et al.* *AtOPR3* specifically inhibits primary root growth in Arabidopsis under phosphate deficiency. *Sci. Rep.* **6**, 24778; doi: 10.1038/srep24778 (2016).

## Supplementary Material

Supplementary Information

## Figures and Tables

**Table 1:** The relative length of the primary root in wild type (WT) and the *Atopr3* mutant plants (*Atopr3*) under the low nitrogen, phosphorus, or potassium condition or under phosphorus deficiency with various chemical additives.

Treatment	WT	*Atopr3*
-N^a^	136.0%	123.2%
-P^a^	57.7%	88.9%
-K^a^	81.3%	84.4%
-P + 1.5 μM JA^b^	89.7%	81.0%
-P + 5 μM IBU^b^	101.9%	124.8%
-P + 5 μM DIECA^b^	106.6%	139.3%
-P + 5 μM AgNO_3_^b^	135.7%	109.0%
-P + 1.25 μM AVG^b^	132.9%	100.7%
-P + 5 μM TIBA^b^	77.0%	67.0%
-P + 7.5 μM GA^b^	135.7%	104.2%
-P + 7.5 μM PAC^b^	76.5%	62.0%
-P + 5 μM Ancy^b^	74.2%	59.5%

^a^indicated comparison of the macronutrient deficient treatment with the full nutrient treatment. “-N”, “-P”, and “-K” represented treatments of low nitrogen, phosphorus, and potassium, respectively.

^b^indicated comparison of the treatment with that without chemical additives under P deficiency.
