# Supplementary material for: AtOPR3 specifically inhibits primary root growth in Arabidopsis under phosphate deficiency
Source: Sci Rep. 2016 Apr 22;6:24778. doi: 10.1038/srep24778 (PMC4840450; doi:10.1038/srep24778)
Supplement: Supplementary Information [file srep24778-s1.pdf]

AtOPR3 specifically inhibits primary root growth in Arabidopsis under phosphate deficiency

Hongyan Zheng, Xiaoying Pan, Yuxia Deng, Huamao Wu, Pei Liu & Xuexian Li*

Figure S1

CTAAACAAACAATGTTTTCTAAAAATGGTATGCCCAAAAATGGTTTACGAAAAATAGACTCAAAAATACAAATGTCATTGGCGAA
AAATAAATGACAATTAATGAATATACAAATAATAGATAAAAAGCAAAATGAAATAAATTTTCAATGTTTCATTTAGTTAAAGATAG
TGTATCTTGAAGATCATAACGTATTTTAGATTATGTACCTGATTTATAAGTGGCTTATAAATCACCTACCATTTTACCAGTAACCGAT
CTAATCTACACACAAATATCATCTTTTACTCTTATAAGATTTTAACCATATTTTTAGATCTCATAAGAATCAATGCAATTTCATATAA
AACATCAAAATAATTCTATAAATTAAAATTTTCTCAAGATAATATTTAATTTTATTTATAAATCTCTTAATTAGCCATCTTTCTACATT
TATCAAAAGATATTAGCTATCTGTTCTCATAGGCAATATGATAAAAAAAAAAACTTTTTCCAAGTTAGGGGCACAAAAAGAGTTT
CCCTGGGACTTGGGCTGAGAATAATGAAGTGGCTGGGAAATGATTAGTTAAGGATGGCTTGATGAATTCTCAATATTCTGTTGAG
GGAACAAAATATACTAGGAAGGATGCAAGTCCAGTTTGGTCTAAACCATTGTACGAACCAAAAACAATCAATAAAGTAACTCAG
GGCTCACAAATGGTCGATAATATTTCTAAAGTTTCAGTTCTAGAGGATGAGCAAAAGGATTTGATGATGGGAGATGTACTAGAGG
TACAAGACAAGAGTCTCACTGAAGGGTTGAATTTCAGATATTTTAAATCACGTTTGGAGTAACAAAAAACAAATGTGAGAAGGA
GCTCAATTAGCTCCAACTGAATCTAGTGTTATTAGGGATACTTTGGAAACTCCAATTCAATCTTTAAGATGTAGGATTGATGAATT
CATGGAAATCGGCAGATCCATCTATGAATTTAGTATGGTGGTGTTTCCTAGGAATTGACAATACAATTTTACTTGGTGCAAAGTGT
CTTCGGAGGTGTCCCTTTCTTTTTCACTCGGAATTGGAAGTTTTAATATTGGCAATAAAAAATATCCTTTCAAGAAGGATAAAGTG
TCAGGCATTTGAAACAGATAATTATTCAGAGTAAGTTTTGATGGTACAGTCCCCTGAAGAATGACTCGCTTTCTCTAATTTTTTAG
AAGATCTTGAATTGCTATGTGTCTCCTTTTCTTGCTTTCTTCTATTTCATGTTTATGTAATACTAAGGCAAACTATTTAGCACAATAT
TCTAAACTTTTATTTTCTGAAGTTATTTTTTAAACCCTTTCCCTCCTAATTGGACAACCAATCATGGAATTAGTTTTTACGTAATGT
TTTAAACTCACTATTTCACCTTTATTCTGACCTGTTAATGGAAGACAACTATGACTTTATACATTGAAATGTTGTCAACCTGTTATA
TATTAATGTTAACATAGCTAATGACCTATCAATGTACGTAAAGCGAATGTCAATCATATTCGTATCCGTTAATTTCATTTAGGAAATC
AGGCTCATTGCCAATCCACTAAGAAACATCTCTGATAGATCCAAATAATAAAAATAATATAATATGAAGCAAAATAGAACGAAATA
TCTCACGCGGACCAAACTTTGAACTTTATTATAAAACAAATCAGCACGTGAAAGTAATGGTCGGTTCGGTCCAAGATGCCAAGA
ACTCCTCCGGTCCATCCATCAACCTAACCTTTTTTGGATATAAAATTTAGCAAATTCCATTTCCACGACCACACAACAACAACACA
TCCACTCGAATTTTCTATTTCCGAATAACAAAACCGGTAAACTCATCACAAACTTAAGTTACTTTGTAGTTTGTGTTTTGTCCTTT
TATGTAATGAAGAAGATCCAACTAACTACTACTCAATATTTTTGTATTGACCGTTGATTTCTCAGATCTGACTTTTACTTCTCCTTC
TTCCAGATCGGCGGAGACATG

Figure S1. The 5'-upstream region of *AtOPR3*.

The putative P1BS element, P1BS-like element, and the start codon were highlighted in red, green, and yellow, respectively.

Figure S2

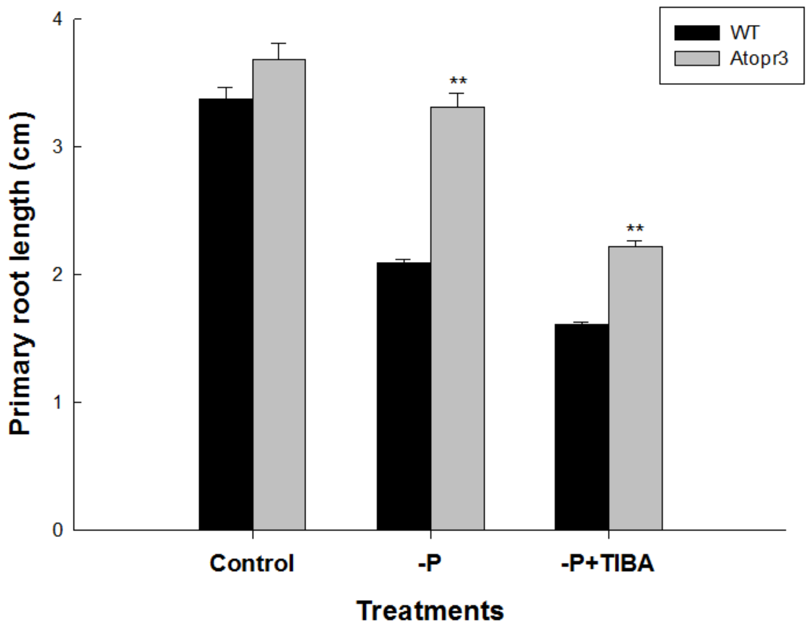

Figure S2. Auxin polar transport and primary root growth in *Atopr3* and WT plants under P deficiency.

Seedlings were grown as described in Fig. 2. TIBA (5μM) was applied to inhibit auxin polar transport. Results were presented as means (n=30) with error bars (standard deviation). Asterisks indicated significant differences as determined by a t-test analysis (**, P<0.01).

Table S1. The relative length of the primary root in wild type *Arabidopsis* (*Ws*) and *opr3* mutant plants (*Atopr3*) without or with various concentrations of chemicals under P deficiency. The root length without the chemical treatment was set as 100%.

**Concentration (μM)**	**GA**		**PAC**		**Ancymidol**		**TIBA**	
	**WT**	***Atopr3***	**WT**	***Atopr3***	**WT**	***Atopr3***	**WT**	***Atopr3***
**0**	100%	100%	100%	100%	100%	100%	100%	100%
**0.5**	-	-	-	-	-	-	-	-
**1**	-	-	-	-	-	-	92.5%<br>-	97.2%<br>-
**1.5**	-	-	-	-	-	-	-	-
**2**	-	-	-	-	-	-	-	-
**2.5**	114.3%	108.0%	70.9%	62.3%	78.9%	76.0%	-	-
**3**	-	-	-	-	-	-	83.1%	83.8%
**5**	116.2%	110.3%	77.0%	64.1%	74.2%	59.5%	77.0%	67.0%
**7.5**	135.7%	104.2%	76.5%	62.0%	66.7%	54.2%	-	-
**10**	100.7%	94.4%	-	-	-	-	-	-

Table S2. Primers used to analyze gene expression levels via RT-qPCR in our studies.

Gene	Primer sequence
AtOPR3	5'—3' AAGGTGTAGTTTCAGCCATAGGA
	5'—3' GATAGTGGGTCAGAATCAGTTGC
AtACS2	5'—3' AGATCGTCGAGAAAGCATCTG
	5'—3' GAAGAGGTGAGTGTGGTGAC
AtCTR1	5'—3' GCCACCCAGTGCTAATATGCC
	5'—3' CAAAGGAACCTGCTCCAATCT
At20OX1	5'—3' GATCCATCCTCCACTTTAGA
	5'—3' GTGTATTCATGAGCGTCTGA
At20OX2	5'—3' ACCGAGACTATTTCCGAGGATT
	5'—3' TGTTTGGCATGGAGGATAATG
At20OX3	5'—3' CCTATCTGCATATGGACTC
	5'—3' AAACCTTCCCGAAATCTTC
At3OX1	5'—3' TCCGAAGGTTTCACCATCACT
	5'—3' TCGCAGTAGTTGAGGTGATGTTG
At3OX2	5'—3' CCTCGCGACTTCTCGAC
	5'—3' AATAATTTCACAGTATTTGAGG
At2OX1	5'—3' CGGGAACTTTCAGAAACGC
	5'—3' ACATTCTTACCACCATTGG
At2OX2	5'—3' CCTAAAACCTCCGCCGTTTT
	5'—3' CCTTCATGTACTCCTCCACCGA
AtLPR1	5'—3' AGAAACTCCAAAATCAGGGACTACA
	5'—3' ATGGTCCAATATGTGACAATGGTAG
ATLPR2	5'—3' GAGGCATAAAGCAAGAGGAGC
	5'—3' GCAGGCAAGTCTCTGTGGAA
ATPRD	5'—3' CAAATGCGCTTCGTATCTCC
	5'—3' TAAAACAGCGTCTGCGTCTG
ATPDR2	5'—3' CTTTTCACTACGTGGTCCGTGG
	5'—3' TGTCTCCCGAGGATGCTGAAT
ATPLD1	5'—3' TGGATGGCAACCGCAAAGACAA
	5'—3' ATCGTTGTGTGTCCCAGCTTCT
ATPLD2	5'—3' TTTGAGGACGGTCCAATTGCCA
	5'—3' ACAACACCGATCTCAGAGTCTCGT
AtTUB4	5'—3' AGAGGTTGACGAGCAGATGA
	5'—3' CCTCTTCTTCCTCCTCGTAC
